# Bacterial motility depends on a critical flagellum length and energy-optimized assembly

**DOI:** 10.1073/pnas.2413488122

**Published:** 2025-03-11

**Authors:** Manuel Halte, Philipp F. Popp, David Hathcock, John Severn, Svenja Fischer, Christian Goosmann, Adrien Ducret, Emmanuelle Charpentier, Yuhai Tu, Eric Lauga, Marc Erhardt, Thibaud T. Renault

**Affiliations:** ^a^Institute of Biology–Department of Molecular Microbiology, Humboldt-Universität zu Berlin, Berlin 10115, Germany; ^b^IBM Thomas J. Watson Research Center, Yorktown Heights, NY 10598; ^c^Department of Applied Mathematics and Theoretical Physics, University of Cambridge, Cambridge CB3 0WA, United Kingdom; ^d^Max Planck Unit for the Science of Pathogens, Berlin 10117, Germany; ^e^Max Planck Institute for Infection Biology, Berlin 10117, Germany; ^f^Molecular Microbiology and Structural Biochemistry, Institut de Biologie et Chimie des Protéines, CNRS UMR 5086, Université de Lyon, Lyon 69367, France; ^g^Univ. Bordeaux, CNRS, INSERM, Acides nucléiques: Régulations naturelles et artificielles, UMR 5320, U1212, Bordeaux F-33000, France

**Keywords:** bacterial flagellum, type-III secretion, cost–benefit tradeoff, macromolecular structures, hydrodynamics

## Abstract

The flagellum is the most complex macromolecular structure known in bacteria and is composed of around two dozen distinct proteins. The main building block of the long, external flagellar filament, flagellin, is secreted through the flagellar type-III secretion system at a remarkable rate of several tens of thousands of amino acids per second, significantly surpassing the rates achieved by other pore-based protein secretion systems. The evolutionary implications and potential benefits of this high secretion rate for flagellum assembly and function, however, have remained elusive. In this study, we provide both experimental and theoretical evidence that the flagellar secretion rate has been evolutionarily optimized to facilitate rapid and efficient construction of a functional flagellum. By synchronizing flagellar assembly, we found that a minimal filament length of 2.5 μm was required for swimming motility. Biophysical modeling revealed that this minimal filament length threshold resulted from an elasto-hydrodynamic instability of the whole swimming cell, dependent on the filament length. Furthermore, we developed a stepwise filament labeling method combined with electron microscopy visualization to validate predicted flagellin secretion rates of up to 10,000 amino acids per second. A biophysical model of flagellum growth demonstrates that the observed high flagellin secretion rate efficiently balances filament elongation and energy consumption, thereby enabling motility in the shortest amount of time. Taken together, these insights underscore the evolutionary pressures that have shaped the development and optimization of the flagellum and type-III secretion system, illuminating the intricate interplay and cost-benefit tradeoff between functionality and efficiency in assembly of large macromolecular structures.

## Significance

Our study demonstrates how protein secretion of the bacterial flagellum is finely tuned to optimize filament assembly rate and flagellum function while minimizing energy consumption. By measuring flagellar filament lengths and bacterial swimming after initiation of flagellum assembly, we were able to establish the minimal filament length necessary for swimming motility, which we rationalized physically as resulting from an elasto-hydrodynamic instability of the swimming cell. Our biophysical model of flagellum growth further illustrates how the physiological flagellin secretion rate is optimized to maximize filament elongation while conserving energy. These findings illuminate the evolutionary pressures that have shaped the function of the bacterial flagellum and type-III secretion system, driving improvements in bacterial motility and overall fitness.

Most bacteria use the flagellum, the largest self-assembling protein structure known in bacteria, as their primary motility device (1). It is produced in consecutive steps that are regulated on multiple levels (2, 3). The hook-basal body (HBB) that contains the flagellar secretion system has a molecular weight of ∼15 MDa in *Salmonella enterica* (4). After assembly of the rod that spans the bacterial cell envelope, the hook is assembled as the first extracellular structure protruding about 55 nm outside of the cell (5). Subsequently, the long filament with a molecular weight of ∼110 MDa per μm self-assembles from a single protein, flagellin. The hook and flagellum, helical and hollow structures, are distinct in their mechanical properties: The hook is supercoiled and flexible, while the flagellum is supercoiled and rigid (6). The rotation of the flagellar motor consisting of the membrane proteins MotAB (stator) and FliG (rotor) in *S. enterica* (7, 8) is transmitted to the rigid filament via the flexible hook that acts as a universal joint. The physics underlying this movement involves the rotation of each motor inducing rotation of the hook and the flagellar filament attached to it, thereby generating propulsion in viscous fluids (1, 9).

While assembly of the bacterial flagellum is well understood from a structural perspective, many questions remain concerning the molecular mechanisms of protein secretion. Most components of the flagellum, including the rod, the hook, and the filament subunits, are substrates of the flagellum-specific type-III secretion system (fT3SS) that is located at the base of the HBB structure (10, 11). Translocation of flagellar substrates across the cytoplasmic membrane depends on the electrochemical gradient of protons (proton motive force, pmf) and is likely mediated via the basal body membrane protein FlhA (12, 13). The external flagellar filament can grow to a length of 10 to 20 μm, corresponding to ∼20,000 to 40,000 flagellin subunits (14). Several models have been proposed to explain the energization of flagellin export and assembly of the filament subunits outside of the cell (12, 15–18). Although the exact mechanism underlying energetics of protein translocation remains unclear, the pmf-dependent pumping activity of the fT3SS results in an injection force, which propels the substrates across the cytoplasmic membrane into the 2 nm wide secretion channel of the flagellum, where they further diffuse to reach their site of assembly at the distal end of the growing structure (14, 15, 19, 20).

Assembly of the bacterial filament itself is surprisingly fast; previous studies, using real-time fluorescence microscopy and stepwise filament fragment labeling, experimentally revealed the kinetics and length dependency of flagellar filament elongation (19, 21). In *S. enterica*, the filament growth rate is initially very fast (∼100 nm/min, corresponding to 3.5 flagellins/s or ∼1,700 amino acids per second) and quickly decreases in a nonlinear way to fall below 20 nm/min. Interestingly, however, the theoretical injection rate of the first flagellin molecule, the *k*_on_ parameter in the kinetic injection diffusion model (19), was estimated to be 30 s^-1^ (or 30 flagellins/s, equivalent to ∼15,000 amino acids per second). Experimental determination of the *k*_on_ parameter is not directly possible by measuring filament elongation using the stepwise labeling approach, since what is measured is an average speed based on increments of the filament length over a defined time, which inevitably underestimates the true parameter. We note that the theoretical secretion rate of the fT3SS is several orders of magnitude higher than that of other protein secretion pores [e.g. 16 amino acids per second in the type-I secretion system (22), 40 amino acids per second in the Sec translocon (23)], raising the question of the biological relevance of such a high secretion rate. The ability to move is of paramount importance for bacteria, as motility provides substantial fitness benefits such as enhanced nutrient acquisition, avoidance of toxic substances, and effective colonization of hosts and surfaces (24–27). It therefore appears reasonable to speculate that enabling fast assembly of the flagellar filament while balancing the associated energy costs of a rapid secretion process would appear beneficial in order to acquire the ability to move as soon as possible.

Here, we combine analyses of single-cell swimming velocities and flagellar filament lengths to reveal a swift transition between nonswimming and swimming bacterial subpopulations at a filament length threshold of ∼2.5 μm. We apply these findings to a biophysical model of swimming bacteria to rationalize how a minimal filament length of ∼2.5 μm is sufficient to enable swimming motility. We further develop an improved filament labeling method using electron microscopy and short elongation time (down to 30 s) to experimentally validate the predicted rapid flagellin secretion rate of ∼10,000 amino acids per second. In order to rationalize the costs/benefits of such a rapid flagellin secretion rate, we develop a biophysical model of flagellum growth that describes the energy costs associated with flagellin secretion with measurements of swimming motility and reveals that the observed rapid flagellin secretion rate is within the range of optimal efficiency.

## Results

### Minimal Flagellar Filament Length Required for Motility.

**Fig. 1.**
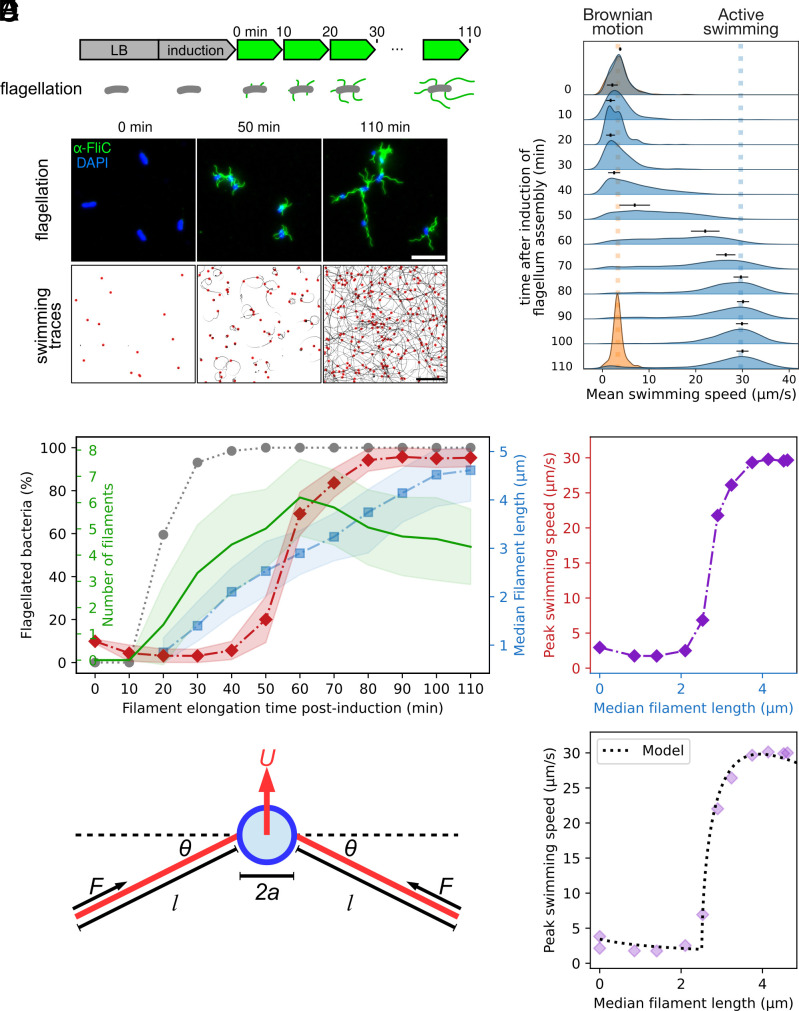
Minimal and optimal flagellar filament length to enable swimming motility. (*A*) Schematic of the experimental setup and exemplary images of the immunostaining for flagellar length measurement (*Upper* panel, scale bar, 10 μm) and swimming speed measurement and trajectories identified using iLastik and TrackMate (*Lower* panel, scale bar, 50 μm.) (*B*) Density plot of mean single-cell swimming speed after induction of flagella synthesis. Blue density plots represent the WT swimming speed at the different timepoints; orange density plot represents the moving speed (only due to Brownian motion) of the nonmotile ΔmotA::mudJ strain at t = 0 min and 110 min after medium switch. The dots and whiskers above the blue density plots indicate the average speed ±1 SD of the 30% most common speed bins in the population. (*C*) Proportion of flagellated bacteria (gray dots); filament length (blue, at least 100 filaments per time point were analyzed); number of filaments (green, at least 100 cells per time point were analyzed) and peak swimming speed (red) as defined in panel (*B*), for different time points after induction of flagellum assembly. The error bands show ±1 SD. (*D*) Peak swimming speed relative to the median filament length indicate a threshold between 2 to 3 μm filament length in order to reach maximal swimming speed. (*E*) Minimal physical model for a peritrichously flagellated bacterium, consisting of a body of radius *a* and flagella of axis lengths *l*. The flagella are active and each generates a parallel driving force *F*. Each flagellum is attached to the body by a torsional spring of spring constant *K* and deflection angle *θ* from the relaxed state. (*F*) Fit of the physical model on the experimental data from panel (*D*) with N = 5 flagella.

Unlike the flagellar rod, hook, and injectisome needle, there is no direct mechanism controlling filament length (28). It is commonly accepted that while flagellar filaments grow up to 15 to 20 μm, a much shorter length (at least one helical pitch, or about 2 μm) is sufficient for bacteria to swim and to allow changing direction (29–31). Thus, it appeared reasonable to assume that long filaments are merely a byproduct of the growth kinetics and that the delay in becoming motile is rather the biologically important parameter. To address this question experimentally, we used an anhydrotetracycline (AnTc) inducible flagellar master regulator (P*tetA-flhDC*) to synchronize flagella biosynthesis (19). This allowed us to simultaneously track flagellar filament growth and observe the swimming behavior of individual bacteria (19) (Fig. 1*A* and *SI Appendix*, Fig. S1). After induction of flagellar gene expression using addition of AnTc, we recorded the fraction of motile bacteria and their swimming speed while determining the lengths of flagellar filaments using immunostaining (Fig. 1*A*). We observed that virtually all bacteria within the population (>90%) were flagellated after 30 min postinduction. Swimming motility was observed after 40 min post induction, and swimming speed sharply increased between 50 and 60 min post induction before reaching a maximal speed of ∼30 μm ${\text{s}}^{-1}$ after 80 min post induction (Fig. 1 *B* and *C*).

As expected, no swimming above background Brownian diffusion was observed in a mutant unable to rotate its flagella (ΔmotA) (Fig. 1*B*). In contrast, for the wildtype (WT), reaching a flagellar filament length of 2.5 μm marked a sharp transition between no motility and the maximal swimming speed; below 2 μm, no swimming was observed among the population (Fig. 1 *C* and *D*). Interestingly, any subsequent increase in filament length did not provide any substantial speed increase, as observed when plotting the filament length across the population relative to the maximal swimming speed measured (Fig. 1*D*).

To rationalize the physical origin of the experimentally observed continuous but rapid transition from no swimming for small flagellar filaments to swimming for flagellar filaments beyond some critical length, we extended a previous physical model of the swimming transition for soft hooks (32). We modeled the cell as a rigid spherical body, to which are attached a number *N* of rigid, force-generating filaments (modeling the action of propelling, rotating flagellar filaments), each connected to the body via a torsional spring to approximate the dynamics of the flagellar hook (33) (Fig. 1*E*). We resolved the force and torque balance on the model bacterium, ultimately obtaining an equation for the stable equilibrium angle *θ* between the axis of each rotary motor and its corresponding flagellar filament; a numerical solution allowed us to identify the stable angle and hence to deduce the corresponding swimming speed of the cell (*SI Appendix*, Data). For a given set of physical parameters, the model invariably predicts the existence of a sharp transition to swimming at a critical flagellar filament length (Fig. 1*F*). Below the critical length, the stable angle is *θ* = 0 and so no swimming occurs. In this regime, the apparent swim speeds are actually the result of displacement due to Brownian motion. Once the flagellar length surpasses the critical length, *θ* sharply increases. Physically, this is when bundling of flagellar filaments is taking place, resulting in swimming of the whole cell and can be understood as an elasto-hydrodynamic instability. Beyond rationalizing the existence of an instability, our model can be used to infer the value of the hook stiffness in swimming bacteria. Many of the geometrical and dynamic parameters in the model are either known or can be determined theoretically (*SI Appendix*, Data). However, the critical flagellar length (*l*_*c*_) and the flagellar propulsive force (*F*) generated by flagella can be determined by fitting the theoretical prediction against the experimental data. The results are shown in Fig. 1*F* for *N* = 5 flagella and were similar for other values of N (*SI Appendix*, Fig. S3). Our theoretical model predicts $l_{c}\approx 2.51\;\mu \;\text{m}$ and F≈1.09pN. In turn, these can be used to deduce the value of the spring constant of the hooks, and consequently their bending modulus, which we find to be $EI\approx 5.2\cdot 10^{-26}\;\;\text{N}\cdot {\text{m}}^{2}$; the model can be further used to calculate the torque generated by the rotating motors and the angular rotation rate of the flagella (*SI Appendix*, Data and Tables S2–S4). We calculate a torque of $1.1\cdot 10^{-18}\;\text{Nm}$, consistent with previous observations (34). Angular rotation rate varies between experiments, depending on flagella length *l* and fluid viscosity *μ*, but generally agrees with previous observations, typically a bit more than 100Hz (34, 35). Notably, the value of *EI* we determined here is smaller than previous in vivo measurements. It has been established that the molecular motor twists the hook, causing changes in its molecular structure which have the effect of increasing its bending modulus *EI* during standard swimming (9, 33, 36). Strikingly, the value of *EI* in our data appears smaller than expected, being associated with very low levels of twist (9, 33). It is possible that the compressive force *F* acting on the hook causes molecular changes which act to reduce the bending modulus in contrast to the twisting, which we see is beneficial for triggering the elasto-hydrodynamic instability necessary for swimming but could also be useful for the purposes of initiating a tumble (33). Another possible explanation is the effects of Brownian motion on individual cells, which could allow the elasto-hydrodynamic instability to occur more easily, which would effectively be similar to a reduction in *EI*. The most notable prediction of our biomechanical model is the existence of a theoretical maximum swimming speed, which coincides with flagellar filament lengths observed experimentally (Fig. 1*F*), suggesting that swimming speed is the parameter that the bacteria choose to optimize.

### The Injection-Diffusion Model Predicts a Narrow Range of *k*_*on*_ for Optimal Filament Elongation.

**Fig. 2.**
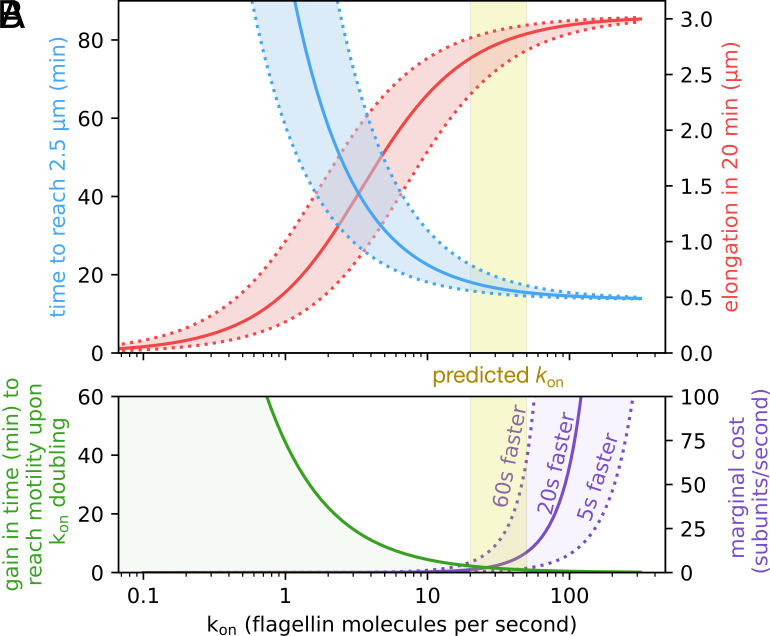
Injection by the fT3SS is a very fast process that is optimized for cellular energy conservation. (*A*) The natural *k*_on_ value (yellow shaded area) is an optimum between fast elongation speed and energy conservation. Filament elongation over about one generation (20 min, in red) and the time required to reach the minimal length suitable for motility (2.5 μm, in blue) increase and decrease, respectively, with *k*_on_. Both values approach the asymptotic extremum for *k*_on_≈ 20 to 50 s^-1^, above which a plateau is reached. The dotted curves show the effect of a ±2 times fluctuation of *k*_on_ around its value. (*B*) Below a few tens of flagellin subunits injected per second, any increase in injection speed significantly reduces the time to reach motility. Above *k*_on_≈ 50 s^-1^, the gain in time drops below a minute. Accordingly, the marginal cost (extra subunits to inject per second to reach motility sooner) significantly increases above this value. This is shown here for a gain in motility onset of 5 s, 20 s, and 60 s. NB. it takes ∼15 to 18 min to reach motility for *k*_on_≈ 20 to 50 s^-1^. The equations for these curves are detailed in *SI Appendix*.

As shown above, we observed that the commonly referenced 20 μm long bacterial filament was not a prerequisite to achieve maximal swimming motility. A short filament was enough to achieve a quasi-maximal function, suggesting that the time needed to reach this threshold length is the physiologically important biological parameter. Accordingly, we next set out to theoretically and experimentally rationalize the effect of the filament elongation rate on the onset of motility. Filament elongation is a nonlinear process driven by two major forces: injection of the flagellins by the export apparatus and diffusion in the hollow filament (19). The injection rate *k*_on_ was previously predicted to be about 30 flagellin subunits per second by fitting the injection-diffusion equation to experimental data obtained by fluorescent multilabeling of elongating flagellar filaments (19). Alternatively, it is also possible to estimate the elongation rate by measuring the length increment over a period of time. In order to rationalize the effect of *k*_on_ on the biological motility parameters, we used the injection-diffusion model to calculate i) the theoretical time to reach the threshold length required for motility, ii) filament length after 20 min of elongation, iii) the gain in time to reach motility upon an increase (doubling) of *k*_on_, and finally iv) the increase in *k*_on_ that would be required to reduce the duration of assembly of a short filament by a few seconds (Fig. 2). All four investigated parameters suggest that optimal filament elongation rate occurs for a *k*_on_ between 20 and 50. For *k*_on_ values below 20, bacteria would have an incentive to spend extra energy in order to achieve motility earlier. For *k*_on_ values above 50, the high injection force does not allow any significant improvement of the elongation kinetics and the incentive is rather to reduce the *k*_on_ to avoid wasting energy (Fig. 2).

### Improving the Estimation of *k*_*on*_ Using Electron Microscopy to Measure the Filament Elongation Speed.

**Fig. 3.**
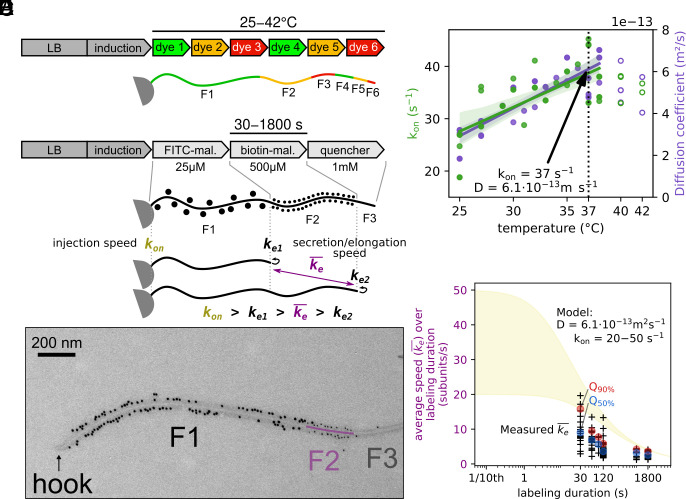
Stepwise high-resolution filament labeling discloses maximal fT3SS injection rate. (*A*) Multilabeling setup of flagellar filaments as previously described in ref. 19. (*B*) The injection rate of the fT3SS (*k*_on_), and the diffusion coefficient of the substrates in the channel (D) increase linearly with temperature up to 38 ^°^C. Above this temperature, heat stress affects secretion. The arrow shows the parameters at the physiological temperature of *S. enterica* (37 ^°^C). (*C*) An improved protocol of stepwise filament labeling allows more precise measurement of filament growth over short periods of time. Use of gold beads of different sizes enables the separation between the basal fragment (F1) and the apical fragment (F2). The average elongation speed $\bar{k_{\text{e}}}$ is necessarily lower than the theoretical *k*_on_. (*D*) Exemplary EM image of a flagellar filament with 60 s staining of the apical fragment. (*E*) Experimentally measured speed of elongation of apical fragment for which the length of the basal fragment F1 is comprised between 0 and 1 μm (143 filaments). Decrease of the average subunit secretion rate is observed as the labeling time increases. The 50th (Q_50%_, in blue) and 90th percentiles (Q_90%_, in red) are shown for each duration of filament elongation and follow the trend of the injection-diffusion model. The quadratic nature of the growth kinetics would require labeling times of less than a second to measure secretion rate close to the theoretical maximum (*k*_on_). The yellow area represents the expected flagellum length for *k*_on_ ≈ 20 to 50 s^−1^.

The injection-diffusion model suggests that an optimal *k*_on_ exists, which reconciles fast elongation and energy efficiency. We next aimed to experimentally determine this value. We first set out to determine the optimal temperature for filament elongation. Employing previously described stepwise filament labeling (19), we determined the growth rate of multilabeled flagellar filaments of bacteria cultivated in a range of temperatures from 25 ^°^C to 40 ^°^C. To facilitate filament length quantification, we developed a method to analyze fluorescently labeled filaments using the Fiji plugin MicrobeJ (*Materials and Methods*). Multilabeling analysis showed an increase in filament growth rate as the temperature increased (Fig. 3 *A* and *B* and *SI Appendix*, Fig. S4). Utilizing the mathematical equation from the injection diffusion model, we plotted the initial injection rate *k*_on_ and diffusion coefficient D across different temperatures. Similar to the growth rate of the filaments, *k*_on_ and D increased from 25 ^°^C to 38 ^°^C. Above 38 ^°^C, a decrease in both parameters can be observed, most likely caused by heat stress (Fig. 3*B*). We therefore performed the next experiments at 37 ^°^C.

The injection-diffusion model predicts a quadratic decrease in filament elongation speed with increasing filament length, which we also observed experimentally using live fluorescent microscopy and stepwise filament labeling (19). In these experiments, we were able to measure an initial elongation speed of ∼3.5 flagellin subunits per second and could estimate a theoretical *k*_on_ of ∼ 30 s^-1^ by fitting the injection-diffusion model. Our stepwise fluorescent labeling protocol allows us to measure an average elongation speed over a defined duration. This value is necessarily lower than the theoretical *k*_on_ value since we measure an average speed over 30 min. In fact, to be able to approach the theoretical *k*_on_ we would need to be able to measure the secretion speed of the first few molecules of flagellin to be assembled, which is not possible with our approach. We, however, estimated that reducing the labeling time to less than a minute could enable us to measure an average elongation speed ($\bar{k_{\text{e}}}$) of about half of the theoretical *k*_on_ value (compared to ∼5 to 10% for 30 min). This required us to improve both the spatial and temporal resolutions over previous stepwise filament labeling methods. Spatial resolution was increased by using electron microscopy (EM) instead of fluorescence microscopy, which allowed us to measure fragments with a precision of a few tens of nanometers (vs. a few hundred nanometers previously). Our previous protocol was based on using thiol-reactive fluorescent dyes to covalently label the flagellin subunits. We performed centrifugation steps to remove the unbound dyes before proceeding to the next fragment labeling. In this study, temporal resolution was improved by optimizing the protocol to remove the centrifugation/washes steps. In brief, we labeled successive fragments with increasing concentrations of two maleimide compounds 1) FITC-maleimide and 2) biotin-maleimide, to label the basal fragment (F1) and the apical fragment (F2) respectively. After labeling the apical fragment, the reaction was stopped using an excess concentration of a quencher (DTT). The increasing concentrations of the labeling molecules ensured an instant transition between fragments (*SI Appendix*, Fig. S5). FITC- and biotin-labeled fragments were immunostained using antibodies coupled with gold particles of different sizes (10 nm and 5 nm, respectively), observable using EM and enabling the separation between apical/basal fragments (*Materials and Methods*). This approach facilitated precise labeling of filaments for durations ranging as short as 30 s to as long as 1,800 s (Fig. 3 *C* and *D*). As we aimed to measure the subunits’ secretion as close as possible to the maximal elongation speed, we analyzed labeled apical fragments for which the basal fragments were in the early stage of assembly, with a length shorter than 1 μm. As predicted, decreasing labeling times revealed much higher flagellin secretion rates than previously measured (Fig. 3*E*). We were able to experimentally measure $\bar{k_{\text{e}}}$ values close to 10 to 20 flagellin molecules per second—almost 5,000 to 10,000 amino acids per second—which is more than 3 to 6 times faster than our previous measurements (19). We note that the 90% quantile of 16 subunits per second is about half of the predicted *k*_on_ value of 37 (Fig. 3*B*), experimentally validating that secretion reaches extremely fast rates in the early stages of filament elongation and suggesting an initial secretion speed of potentially several tens of thousands of amino-acids per second. We hypothesize that the observed variability in average speed for a given elongation time is due to the cell to cell biological variability in the available pmf and cytoplasmic pools of flagellins as previously observed (37).

### The Microscopic Mechanism of Inserting a Flagellin Molecule in the fT3SS Secretion Channel and the Energy Cost for Achieving an Injection Rate *k*_*on*_.

To understand the biological implications of the observed rapid flagellin secretion rate, we next developed a biophysical model of flagellum growth, which correlates the energy costs of flagellin secretion with our swimming motility measurements. In this model, the injection rate *k*_on_ represents the speed at which a partially unfolded flagellin is fully inserted into the channel. This process involves two primary steps: i) The partially unfolded flagellin (FliC) arrives at the entry point of the basal body; ii) The flagellin is inserted segment-by-segment into the channel by a pmf-powered secretion system located at the basal body of the flagellum. Together the arrival and insertion timescales, *t*_*a*_ and *t*_*i*_, determine to the overall injection rate, $k_{\text{on}}={\left(t_{a}+t_{i}\right)}^{-1}$. We have developed microscopic models for the mechanisms underlying both arrival and insertion steps of the injection process, which allow us to estimate the associated time scales and energy costs (full details in *SI Appendix*, Data). Our results provide insights into how the bacteria can improve their motility by spending more energy to increase the injection rate *k*_on_ and show that the experimentally observed range of *k*_on_ lies within the range of optimal efficiency.

**Fig. 4.**
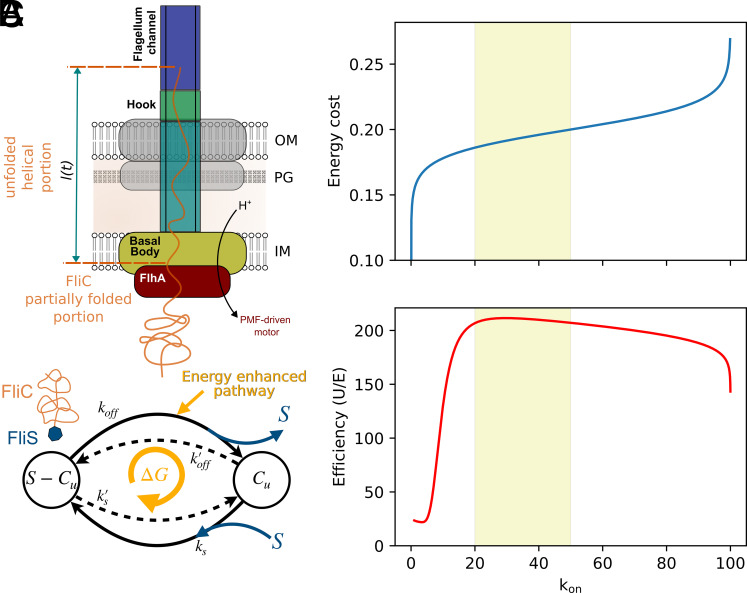
Model for the energy cost of flagellin secretion. (*A*) The energy–rate relation for the flagellin injection process via the fT3SS. The schematic illustrates the active injection process powered by a pmf-driven motor and a minimal model for the flagellin arrival at the export gate. We consider two primary states for flagellin: partially unfolded FliC bound to FliS and free FliC available for export. Transitions between these states involve association/dissociation between chaperone and substrate in solution (with rates $k_{s}\gg k_{s}^{\prime }$) and a second pathway that strips off the chaperone via energy consuming interactions with the ATPase and/or a pmf powered mechanism. (*B*) The energy cost per arrival *E*_*a*_ predicted by our model and (*C*) efficiency η=U/E plotted as a function of the resulting injection rate *k*_on_. Here, the utility function *U* is the measured average swimming speed as a function of flagella length. Increasing the injection rate requires larger energy cost that is used to increase the local concentration of FliC near the export gate. This combined with the diminishing returns for swimming speed leads to a peak efficiency within the shaded range corresponding to experimentally measured injection rates, $k_{\text{on}}\approx 20\;\text{to}\;50\;{\text{s}}^{-1}$.

The insertion step involves balance between the pmf, which pushes the flagellin into the channel segment-by-segment, and an entropic force that tends to push the flagellin out of the channel (Fig. 4*A*). The later force arises because the fully extended flagellin in the channel is a high entropy state compared to outside the channel where the unfolded molecule has access to many more configurations. The insertion speed is determined by these forces along with the drag coefficient *μ*_0_ per unit length of inserted flagellin molecule. To estimate this, we use the Einstein relation, $\mu_{\text{tot}}=\mu_{0}L=k_{\text{B}}T/D$, where *L* = 74 nm is the total extended length of the flagellin (38, 39), *k*_B_ is Boltzmann’s constant, *T* is temperature, and $D=6\cdot 10^{-13}{\text{m}}^{2}{\text{s}}^{-1}$ is the diffusion constant in the channel, which is fitted by using the injection-diffusion model and the flagellar filament elongation rate measurements as described in the preceding sections.

We find that the drag coefficient is very small, $\mu_{0}=\left(2.3\cdot 10^{-8}\;{\text{s/nm}}^{3}\right)k_{\;\text{B}}T$. For typical pmf energy scale 5 to 10 $k_{\text{B}}T$ and assuming the flagellin is inserted in segments of 1 to 2 nm, this leads to a total insertion timescale of $t_{i}\approx 10^{-4}$ s (*SI Appendix*, Data). Comparing to the experimental estimates above, $k_{\text{on}}\approx 20\;\text{to}\;50$ s^−1^, we see that insertion is not the dominant timescale and therefore is not a useful target for improving performance. To summarize, it is important that the pmf can overcome entropic forces that tend to push a partially inserted flagellin out of the channel, but as long as the pmf is sufficiently strong the insertion will be extremely fast.

Given the above analysis, the arrival step is therefore rate-limiting for determining the overall injection rate *k*_on_, which is consistent with previous observations of pauses in filament elongation, attributed to insufficient cytoplasmic flagellin supply (37). Association with the chaperone FliS on its C-terminal disordered region leads FliC to be directed to the secretion pathway (40, 41). The chaperone is stripped off before insertion, which incurs an energy cost. This had been thought to be mediated by ATP hydrolysis by the ATPase complex, but mutants without ATPase or suppressed ATPase activity are able to export FliC (15, 16). It is therefore plausible that the pmf is at least partially responsible for chaperone removal, perhaps via interactions with the FlhA ring before the unfolded and unchaperoned flagellin reaches the export gate. Because of this uncertainty in the microscopic mechanisms, we employ a simplified but general two-state kinetic model pictured in Fig. 4*A*. In solution, flagellin heavily favors binding to FliS with association and dissociation constants *k*_*s*_ and $k_{s}^{\prime }$ respectively ($k_{s}\gg k_{s}^{\prime }$). A dissipative mechanism spends ΔG free energy per cycle to enhance a second reaction pathway that strips off FliS (with rate *k*_off_), leaving an unfolded flagellin molecule, which can either be exported or rebind to FliS. By spending more energy driving this chemical cycle, the bacteria can increase the local concentration of unfolded flagellin, ${\left[C\right]}_{u}$, near the export gate to decrease the arrival time. Assuming the arrival is a diffusion-limited process, the arrival time is $t_{a}={\left(4\pi dD_{c}{\left[C\right]}_{u}\right)}^{-1}$, where *D*_*c*_ is the diffusion constant for the unfolded flagellin and *d* is the interaction length scale with the pmf export complex (how close the molecule must diffuse to guarantee it is exported). Replacing ${\left[C\right]}_{u}$ the total intracellular concentration of FliC gives a lower bound $t_{a}>t_{\text{min}}\approx 0.01$ s, which is only about three times smaller than the measured $k_{\text{on}}^{-1}$. Using our kinetic model, we next compute the energy dissipated per flagellin export, *E*, and express this in terms of the resulting injection rate *k*_on_ (*SI Appendix*, Data). As shown in Fig. 4*B*, increasing *k*_on_ requires a greater energy cost with diminishing returns: Infinite dissipation is required to push toward the limiting value $k_{\text{on}}=t_{\text{min}}^{-1}$. In the opposite limit, a finite, but very small, *k*_on_ can be achieved with no energy cost due to spontaneous unbinding of the chaperone. Finally, combining this energy cost with our measurements of swimming motility, we compute the efficiency, defined as η=U/E, at which the bacteria spend energy for enhancing flagellum function (Fig. 4). For the utility function, *U*, we use the peak swimming speed as a function of filament length. Due to the combination of increasing energy cost to increase *k*_on_ and the diminishing returns in utility once the flagellar filament elongation rate is sufficiently large, the efficiency peaks around $k_{\text{on}}=20\;\text{to}\;50$ s^−1^; indicating that the experimentally estimated value may be tuned toward optimal efficiency.

## Discussion

Flagellum assembly in bacteria is a tightly controlled process that involves a remarkably fast secretion rate of thousands of amino acids per second through the fT3SS, a pmf-dependent mechanism that is considerably more rapid than the general protein secretion system (Sec system) which secretes only a few dozen amino acids per second and any other known pore-based protein secretion system (23, 42). The physiological importance of such a rapid protein secretion rate has remained unclear. However, it appeared reasonable to speculate that flagellin secretion rate was evolutionary optimized to balance the energy costs of the secretion process and to minimize the time needed to achieve motility. Indeed, when we modeled various biologically relevant parameters for different values of the flagellin secretion rate (*k*_on_), we identified a critical *k*_on_ threshold between 20 and 50 s^-1^. Beyond this threshold, increasing *k*_on_ did not further enhance elongation speed or decrease filament assembly time, but significantly increased energy costs, suggesting an evolutionary optimization of the flagellin secretion rate to reach the required minimal filament length for motility without excessive energy expenditure (i.e. consumption of the proton gradient and the production of flagellin). We therefore determined experimentally the minimal filament length needed for motility after induction of flagellar synthesis. Although the flagellar filament can grow to a length of up to 20 μm, we observed that a length slightly exceeding a critical value in the 2 to 3 μm range was sufficient to attain the maximum swimming speed (Fig. 1). We note that this result is consistent with previous observations that a single helical pitch of the flagellar filament, approximately 2 μm in length, was sufficient to enable bacterial motility (29, 30). Our biophysical model, which effectively simulates the complex biomechanics of flagellar propulsion, reveals that the emergence of swimming at a specific filament length can be attributed to an elasto-hydrodynamic instability of the swimming cell with flexible flagellar hooks. This phenomenon illustrates a mechanical interplay between the hook’s elasticity, aligning the flagellar filaments with the motor axis, and the external hydrodynamic moments induced by swimming flows. The short filaments initially resist these external torques due to the stabilizing elasticity of the hooks. However, as the filaments elongate, they reach a critical length where the external torques overpower the hook’s resistance, thereby enabling swimming.

To understand the energy cost of filament growth, we developed a microscopic model for flagellin insertion into the secretion channel. Two critical steps delineate the injection process characterized by *k*_on_: the arrival of the partially unfolded flagellin and its segment-by-segment insertion, influenced by both pmf and entropic forces. We found that the rate-limiting step is the arrival of unfolded FliC, indicating that this step is a better target for spending energy to increase the overall injection rate. The total energy cost for each cycle of flagellin export, combined with the experimental measurements of swimming motility, allowed us to determine an optimal efficiency peak for the injection rate *k*_on_, reflecting a finely tuned balance that maximizes bacterial motility with minimal energy expenditure. This optimized behavior may reflect evolutionary adaptations that enhance bacterial fitness during swimming motility.

Within the T3SS, one notable instance of optimization is the tightly regulated secretion process that ensures the correct order of component assembly. The T3SS must secrete the rod, hook, and filament proteins in a sequential order to correctly form a functional flagellum. In other words, the T3SS is optimized to recognize and prioritize the secretion of different proteins based on the stage of flagellar assembly (43). The optimization process of the fT3SS can also be observed in the balance between the assembly speed and energy burden. Our work suggests that the fT3SS injection rate has been evolutionarily optimized to strike a balance between efficient flagellum assembly and minimal energy expenditure. This finely tuned balance underlines the intricate evolutionary adaptations that bacteria have developed to enhance their motility and fitness in their specific environments. Our findings also raise questions about the secretion capabilities of virulence-associated type-III secretion systems. If a high injection rate is possible for the flagellar T3SS, it would be interesting to investigate whether similar rates can be achieved in the secretion systems responsible for assembling virulence factors and effectors. A previous study reported a secretion rate of 5 to 60 molecules per seconds of the fT3SS evolutionary-related vT3SS injectisome effector SipA in eukaryotic cells during infection, which is in range with the secretion rate of the flagellin measured during our observation. However, it remains unclear if those numbers result in the secretion of one or several vT3SS attached to the eukaryotic cell (44). Further, investigation of the observed temperature-dependent effects on secretion rate via the T3SS and the secretion kinetics of other secretion systems could yield important insights into how bacteria have evolved to optimize their secretion systems for survival in various environments and host interactions. By decreasing filament labeling to 30 s, we could measure an elongation speed $\bar{k_{\text{e}}}$ of up to 20 s^−1^ half of the theoretical *k*_on_. Stepwise labeling however remains intrinsically limited by temporal resolution and decreasing labeling below a few seconds would be technically challenging. Furthermore, and although not the focus of this study, precise quantification of elongation may also be influenced by factors such as cytoplasmic flagellin availability or membrane potential, consistent with previous observations in *Escherichia coli* where assembly alternates between elongation and pause phases (37). Future advancements enabling simultaneous measurement of elongation rates and these factors will be essential for a comprehensive understanding of fT3SS secretion dynamics.

In conclusion, the findings presented in this study suggest that flagella assembly in bacteria has evolved to enable a swift onset of motility, crucial for survival in changing environments. The bacterial flagellum therefore represents a paradigm example of evolutionary adaptation. Its assembly requires a finely tuned regulation, balancing assembly speed and energy expenditure, thereby showcasing the refinement process of biological optimization.

## Materials and Methods

### Strains, Media, and Growth Conditions.

Strains and genotypes are listed in *SI Appendix*, Table S1. *S. enterica* serovar Typhimurium LT2 was grown in LB (Lennox) (10 g/L tryptone, 5 g/L yeast extract, 5 g/L NaCl) and at 30 ^°^C unless stated otherwise. For protocols requiring washing steps, phosphate-buffered saline (PBS) (8 g/L NaCl, 0.2 g/L KCl, 1.15 g/L Na2HPO4, 0.2 g/L KH2PO4, pH 7.3) was used.

### Swimming Behavior and Filament Immunostaining.

Overnight cultures were incubated in LB at 30 ^°^C and 180 rpm. Subcultures were inoculated 1:100 in 10 mL fresh LB and cultivated accordingly. After 2.5 h of growth, *flhDC* expression was synchronized by induction with AnTc, (final concentration = 100 ng/ml) followed by 30 min of incubation. Subsequently, cells were harvested at 2,500 × g for 5 min and resuspended in fresh AnTc-free media. Directly, at 0 min post medium switch a first sample was drawn and processed for obtaining swimming behavior and filament immunostaining. Cells were placed back for incubation, and new sample acquisition was performed every 10 min up to 110 min post medium switch. For each sample at the indicated timepoints, cells were diluted with LB (1:20 or at timepoints beyond 40 min post medium switch 1:40) to keep cell density below OD_600_ = 0.1. For obtaining swimming behavior of the cells, 70 μL were loaded in a flow cell and microscopy was performed using a Ti-2 Nikon inverted microscope equipped with a CFI Plan Apochromat DM 20× Ph2/0.75 objective. For each timepoint, two positions were monitored for 100 frames with a time interval of 43 ms between frames. Image analysis and tracking of cells was performed using ilastik (45) and Fiji (46) equipped with TrackMate (47). Mean swimming speed was measured with TrackMate and peak swimming speed was defined as average speed of the 30% most common bins in the population. 261 to 655 traces were recorded for timepoints 0 to 30 min and 2,362 to 15,215 traces for timepoints 40 to 110 min. The LoG detector settings were set to three microns and a threshold of 20, combined with the autofunction. For the simple LAP tracker, the maximal linking distance was set to two as well as the values for maximal gap-closing distance and frames. Then, a minimal displacement of three microns was determined as threshold distinguishing between movement and drift. For each timepoint, cells were also prepared for subsequent immunostaining as described previously (48). In brief, poly-L-lysine-coated coverslips were flushed with 100 μL cells followed by fixation with 4% paraformaldehyde for 10 min. All steps were performed at room temperature. After that, cells were washed with PBS and blocked with 10% BSA for 10 min. Primary antibody (anti-FliC, rabbit, 1:1,000 in 2% BSA) was added and incubated for one hour. Subsequently, cells were washed and blocked again as described above. Secondary antibody (anti-rabbit Alexa Fluor488, diluted 1:1,000 in PBS) was added and the mix was incubated for another 30 min. Finally, cells were washed twice with PBS and Fluoroshield with DAPI mounting medium (Sigma-Aldrich) was added. Fluorescence microscopy was carried out using a Ti-2 Nikon inverted microscope equipped with a CFI Plan Apochromat DM 60× Lambda oil Ph3/1.40 (Nikon) oil objective. Microscope settings were set to: 488 nm: 200 ms 2% 16-bit Z-stack every 0.3 μm range 2 μm 9 slides 405 nm: 50 ms 20% 16-bit single plane PC: 100 ms 80% DIA 16-bit single plane. Image analysis (filament counting and length determination) was performed using Fiji (46) equipped with the MicrobeJ plugin using the ROI Manager for measurement of the length (49).

### Temperature Variation and Multilabeling of the Flagellar Filament.

Filament multilabeling was performed as described previously (19) and as shown in *SI Appendix*, Fig. S6. A FliC_*T*237*C*_ cysteine replacement mutant was grown overnight in LB at 30 ^°^C, diluted 1:100 into 10 mL fresh LB in a 100 mL flask and grown at 30 ^°^C for 1.5 h until reaching early exponential phase. P*tetA-flhDC* promoter was induced by addition of AnTc (100 ng/mL) for 30 min. Afterward, cells were collected by centrifugation for 3 min at 9,000 × g, resuspended in 10 mL fresh LB. Aliquots of the culture in 1.5 mL tubes were then incubated for 30 min at the corresponding temperature in a thermomixer with low agitation (300 rpm). Labeling with maleimide dyes up to 6 filament fragment was performed successively as described previously (19). After the final labeling period, cells were resuspended in PBS and 100 μL were applied to a custom-made flow cell coated with poly-L-lysine. Cells were fixed by addition of 4% PFA for 10 min, followed by a washing step with PBS. Fluoroshield with DAPI mounting medium (Sigma-Aldrich) was added and the cells were observed by fluorescent microscopy using a Zeiss Axio Observer Z1 microscope at 100× magnification. A Z-stack was applied for every image to ensure observation of the whole filament. Fluorescence images were processed using Fiji (46) and a custom Fiji macro to fuse the Z-stack.

### Filament Multilabeling Analysis.

Sequentially labeled flagella were detected and analyzed using MicrobeJ 5.13n (49) a plugin for ImageJ (46). Briefly, rough estimations of medial axes of flagella were manually drawn on a maximum intensity channel-projection image using the segmented-line selection tool of ImageJ. The resulting selections were then stored in the ROI Manager and imported in MicrobeJ using the Filament detection mode. The medial axis of each filament was then refined using a transversal local maximum neighbor algorithm and used to compute the geometrical and topological properties of the filament, such as its length, width, sinuosity, curvature, and angularity. The analysis of the fluorescence intensities along the medial axis of each filament was subsequently performed using the Feature option called *Multisection* in MicrobeJ. Briefly, the fluorescent profiles were extracted along the medial axis of the filament for each specified channel and filtered using a window moving average filter. To accommodate global variations of fluorescence intensity between channels, fluorescent profiles were weighted using specific factors for each channel. Sections were defined as the brightest sections of each channel. The relative localization, the length, and the relative order along the corresponding filament were determined for each section (*SI Appendix*, Fig. S6).

### High-Resolution EM Stepwise Filament Labeling.

The previously described protocol (19) was modified as follows. Overnight cultures were incubated in LB at 37 ^°^C and 180 rpm. Subcultures were inoculated 1:100 in 10 mL fresh LB and cultivated accordingly. Cells were grown until an OD600 of 0.6. Expression of *flhDC* was synchronized by induction with AnTc (final concentration = 100 ng/ml) followed by 30 min of incubation. Subsequently, cells were harvested at 2,500 x g for 3 min and resuspended in fresh AnTc-free media. To allow completion of hook-basal body assembly, cells were incubated for further 30 min at 37 ^°^C. The first and second fragments were labeled by successive addition of 25 μM Fluorescein-5-Maleimide (Thermo Fisher Scientific) and 500 μM Maleimide-PEG2-Biotin (Thermo Fisher Scientific), respectively. Labeling reactions were performed at 37 ^°^C and mild agitation. At the end of the second fragment labeling, the reaction was stopped by addition of 1 mM of DTT. The step increase in concentrations was sufficient to precisely determine the limits of the measured fragments. The flagella were then detached from the cell by 10 passes through a 23-gauge needle and the cells were removed by 10 min centrifugation at 3,000 × g. The labeled filaments were applied to carbon and pioloform-film-coated gold grids. FITC- and biotin-labeled fragments were immunostained using anti-fluorescein (Aurion) and anti-biotin (British Biocell) antibodies coupled to 10 nm and 5 nm gold particles, respectively. Of note, due to slight chemical differences in fluorescein and FITC structures, the immunostaining efficiency for the first fragment and thus the gold coverage was not fully optimal, which had no impact on the precision of the second fragment measurement. The samples were acquired on a Zeiss LEO 906 operated at 100 kV and images were recorded with a digital camera. Only intact filaments with both the hook attached and three fragments (10 nm labeled, 5 nm labeled, and unlabeled) were used for the quantification. Fragment length was measured using Fiji (46) and the NeuronJ plugin (50).

## Supplementary Material

Appendix 01 (PDF)

## Data Availability

All study data are included in the article and/or *SI Appendix*.
