# Supplementary material for: Bacterial motility depends on a critical flagellum length and energy-optimized assembly
Source: Proc Natl Acad Sci U S A. 2025 Mar 11;122(11):e2413488122. doi: 10.1073/pnas.2413488122 (PMC11929379; doi:10.1073/pnas.2413488122)
Supplement: Supplementary file 1 — Appendix 01 (PDF) [file pnas.2413488122.sapp.pdf]

Supplementary Information

Bacterial motility depends on a critical flagellum length and energy-optimized assembly

Manuel Halte [a], **Philipp F. Popp** [a], **David Hathcock** [b,1], **John Severn** [c,1], **Svenja Fischer** [a,d], **Christian Goosmann** [e], **Adrien Ducret** [f], **Emmanuelle Charpentier** [d], **Yuhai Tu** [b], **Eric Lauga** [c], **Marc Erhardt** [a,d] ✉, and **Thibaud T. Renault** [a,d,g] ✉

[a]Institute of Biology – Molecular Microbiology, Humboldt-Universität zu Berlin, Philippstr. 13, 10115 Berlin, Germany; [b]IBM Thomas J Watson Research Center, Yorktown Heights, NY 10598, United States; [c]Department of Applied Mathematics and Theoretical Physics, University of Cambridge, Wilberforce Road, Cambridge CB3 0WA, United Kingdom; [d]Max Planck Unit for the Science of Pathogens, Charitéplatz 1, 10117 Berlin, Germany; [e]Max Planck Institute for Infection Biology, Charitéplatz 1, 10117 Berlin, Germany; [f]Molecular Microbiology and Structural Biochemistry, CNRS UMR 5086, Université de Lyon, 7 passage du Vercors, 69367 Lyon, France; [g]Univ. Bordeaux, CNRS, INSERM, ARNA, UMR 5320, U1212, F-33000 Bordeaux, France; [1]D.H and J.S. contributed equally to this work.
Correspondance: thibaud.renault@cnrs.fr and marc.erhardt@hu-berlin.de

doi:10.1073/pnas.2413488122

Supplementary figures

Figure S1 Experimental setup of the fT3SS assembly synchronization in the cell population. 1
Figure S2 Combination of short and long filament population. 1
Figure S3 Fit of the physical model of Figure 1F for different values of N 2
Figure S4 Analysis of multilabeled filaments at different temperatures. 3
Figure S5 Increasing concentrations of molecules enable a wash-free stepwise labeling. 3
Figure S6 MicrobeJ analysis of the multilabeled flagellar filament. 4

Supplementary tables

Table S1 List of strains used in this study 4
Table S2 Key constant physical parameters used in the swimming model. 10
Table S3 Key physical variables of the swimming model. 10
Table S4 Key physical results of the swimming model. 10

Models

Elastohydrodynamic instability of a peritrichous swimmer 5
Predictions for the optimal range of k_{on} from the injection-diffusion model 6
Arrival and insertion timescales and the associated energy cost 8

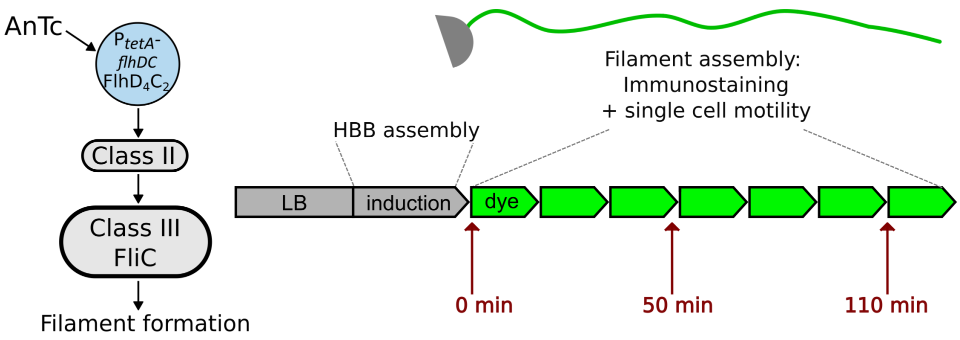

Figure S1. Experimental setup of the fT3SS assembly synchronization in the cell population.
After growth in LB medium, the master regulator *flhDC* expression was induced by addition of AnTc for 30 min, synchronizing expression of *flhDC* followed by class II and class III and the assembly of hook basal body (HBB) and filaments in the bacterial population. After induction, cells were resuspended in LB-medium free of AnTc inducer. Samples were probed every 10 min from the culture, simultaneously filament stained (immunostaining, rabbit α-FliC primary antibody, mouse α-rabbit secondary antibody coupled to Alexa488) and monitored for swimming speed (see Material and Methods).

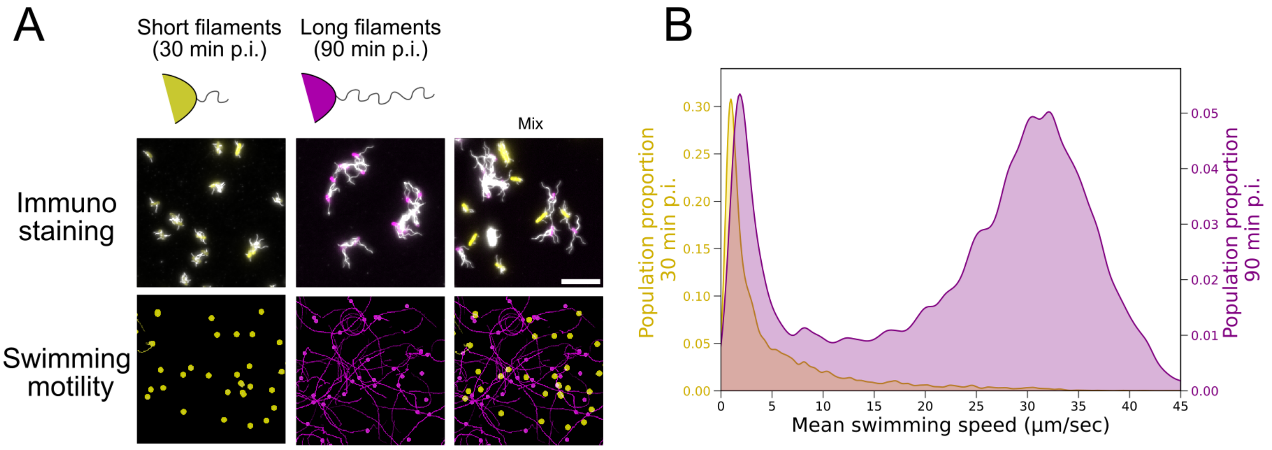

Figure S2. Combination of short and long filament population shows the requirement for a longer filament to display swimming behaviour.
(**A**) Representative image of cells with short/long filament. Population were grown in two different flasks and induced with AnTc as described in Materials and Methods. Either 30 min or 90 min post induction, samples were collected and imaged for swimming speed and filament immunostaining. The short filament population carried a vector constitutively expressing the fluorescent protein mNeonGreen (yellow), while the long filament population expressed mCherry2-L (magenta). Those distinct fluorophores enabled us to distinguish between the 2 populations. Upper panel display the filament immunostaining experiment performed on single population or after mixing the 2 populations. Flagellar filaments were stained with Alexa647-conjugated secondary antibodies (white, scale bar = 10 µm). The lower panel display the swimming experiment performed on mixed population as described in Materials and Methods, except that a Trigger Acquisition module enabled us to track the 2 population in the same slide, with an interval of 40ms between each frame for each fluorescence channel. (**B**) Density plot of the swimming speed for each populations. As observed, the short filament population (yellow) is primarily non-motile or exhibits minimal motility while the long filament population reached the maximal swimming speed observed in Figure 1B.

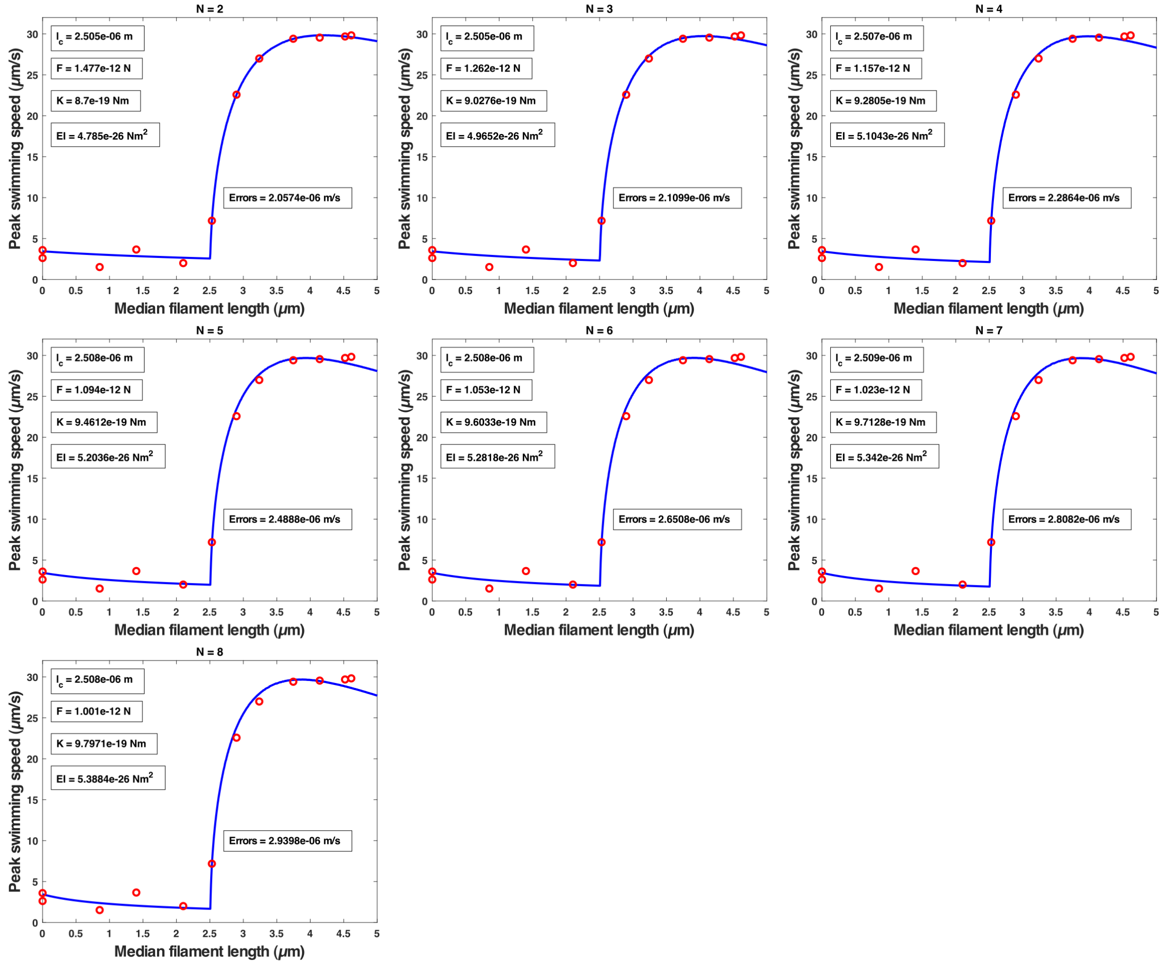

Figure S3. Fit of the physical model of Figure 1F for different values of N (from N = 2 to 8)

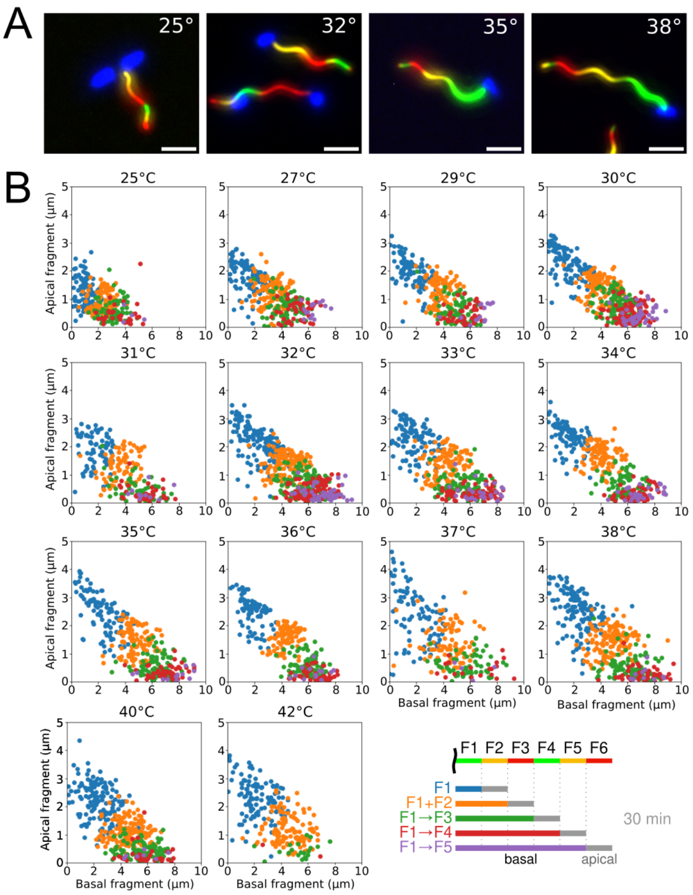

Figure S4. Analysis of multilabeled filaments at different temperatures.
(**A**) Exemplary microscopy pictures of multilabeling experiment, as previously described in (1) (scale bar = 2 µm). (**B**) Exemplary graphs of multilabeled filaments at the different temperatures. Analysis was performed using MicrobeJ plugin as described in Material and Method. At least 100 filaments were analysed per experiment. Values were represented using a custom Python script.

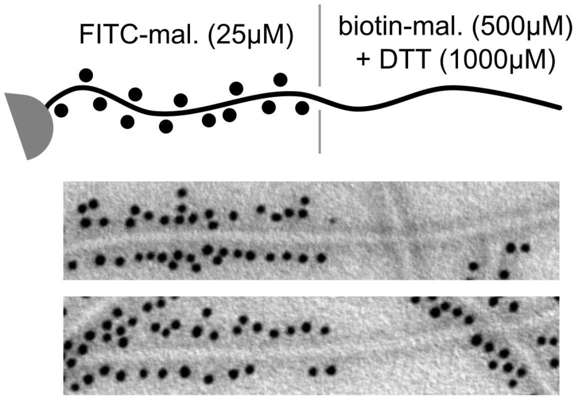

Figure S5. Increasing concentrations of molecules enable a wash-free stepwise labeling.
In order to validate the effect of increasing concentrations of labeling molecules to allow labeling of multiple fragments without needing to exchange the medium, we first labeled the filaments with 25 µM FITC-maleimide, then we simultaneously added biotin-maleimide (500 µM) and DTT (1000 µM). The filaments were then immunostained with anti-FITC (coupled to 10nm gold particles) and anti-biotin (coupled to 5nm gold particles) antibodies. Almost no biotin labeling was observed confirming that the most concentrated compound outcompetes the others.

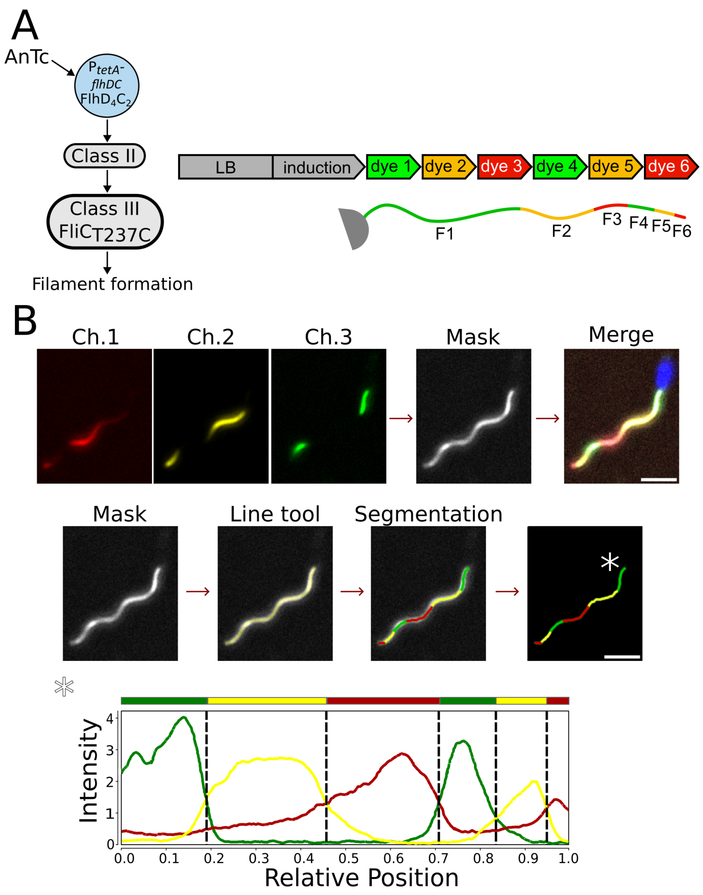

Figure S6. MicrobeJ analysis of the multilabeled flagellar filament.
(**A**) Experimental setup shown as described in the Material and Methods and in (2). (**B**) The multi-labelled flagellar filaments were imaged successively on the red (Ch. 1), the yellow (Ch. 2), and the green (Ch. 3) channels. To facilitate the detection of the whole filaments, the channels were combined into a maximum intensity channel-projection image (Mask) using the "Combine channels" tool of MicrobeJ. The medial axis of each filament was manually drawn on the mask using the segmented line tool and then refined using a transversal local maximum neighbor algorithm. Alternatively, the blue channel was used to detect the cell attached to the filament and to define which end of the filament is connected to the cell body (∗). The oriented fluorescent profiles were then extracted along the medial axis of the filament for each specified channel and used to segment the filament into sections (bottom graph). Sections were defined as the brightest sections of each channel. The relative localization and the length were determined for each section and represented on the filament overlay using their corresponding color.

Table S1. List of strains used in this study

Strain	Genotype	Source
EM4869	Δhin-5717::FRT PtetAflhDC5451::Tn10dTc[del-25]	Lab collection
EM11996	Δhin-5717::FRT PtetAflhDC5451::Tn10dTc[del-25] ΔmotA5461::mudJ	This study
EM2046	Δhin-5717::FRT PtetAflhDC5451::Tn10dTc[del-25] fliC6500 (T237C)	Lab collection
EM18110	Δhin-5717::FRT PflhDC5451::Tn10dTc[del-25] / pEM8315 (pKH70-PrpsM-mCherry2-L, AmpR)	This study
EM18111	Δhin-5717::FRT PflhDC5451::Tn10dTc[del-25] / pEM8731 (pKH70-PrpsM-mNeonGreen, AmpR)	This study

Model: Elastohydrodynamic instability of a peritrichous swimmer

Governing equations. The parameters used in the following equations are defined in Tables S2–S4).
A global force balance on the system gives

$$\frac{12\pi\mu a U}{N} + 2lU\left(C_{\parallel}\sin^2(\theta) + C_{\perp}\cos^2(\theta)\right) - \dot{\theta}l^2 C_{\perp}\cos(\theta) = 2F\sin(\theta), \quad \textbf{(E1)}$$

whilst a torque balance on each flagellum gives

$$\frac{l^3}{3}C_{\perp}\dot{\theta} - \frac{l^2}{2}UC_{\perp}\cos(\theta) + K\theta = 0. \quad \textbf{(E2)}$$

These equations can be combined for an evolution equations for θ. By setting $\dot{\theta} = 0$ we obtain the equilibrium equation which gives the value of θ for given flagellar length l:

$$K\theta = \frac{Fl^2 C_{\perp}\sin(\theta)\cos(\theta)}{\frac{12\pi\mu a}{N} + 2l\left(C_{\parallel}\sin^2(\theta) + C_{\perp}\cos^2(\theta)\right)}. \quad \textbf{(E3)}$$

Clearly, $\theta = 0$ is always an equilibrium angle. However, defining a critical length l_c implicitly as

$$K = \frac{Fl_c^2 C_{\perp}}{\frac{12\pi\mu a}{N} + 2C_{\perp}l_c}, \quad \textbf{(E4)}$$

the evolution equation can be perturbed about $\theta = 0$ to show that $\theta = 0$ is stable if and only if $l < l_c$. When $l > l_c$, a new stable equilibrium at $\theta > 0$ is created. Whether $l < l_c$ or $l > l_c$, assuming the system has reached its stable equilibrium, the swimming speed can then be calculated as

$$U = \frac{2F\sin(\theta)}{\frac{12\pi\mu a}{N} + 2l\left(C_{\parallel}\sin^2(\theta) + C_{\perp}\cos^2(\theta)\right)}. \quad \textbf{(E5)}$$

Physical parameters. Before we can solve the governing equations, we must identify appropriate values for various physical parameters, and justify certain assumptions.

The helical pitch angle of the flagella is approximately $30°$ (3, 4), so for an axial length l, the contour length is given by $L = 2l/\sqrt{3}$. This provides appropriate definitions for the Resistive Force Theory drag coefficients

$$c_{\parallel} = \frac{2\pi\mu}{\log(2l/r\sqrt{3}) - 1/2}, \quad \textbf{(E6)}$$

$$c_{\perp} = \frac{4\pi\mu}{\log(2l/r\sqrt{3}) + 1/2}, \quad \textbf{(E7)}$$

where $r = 12\,\mathrm{nm}$ (4) is the contour radius and μ is the dynamic viscosity of the fluid. These serve as expressions for the fluid force per unit length exerted on a slender filament of length $2l/\sqrt{3}$ travelling at unit speed. However, the helical geometry of the real flagella complicates this. Integrating for the fluid force along the length of the contour gives total fluid forces

$$F_{\parallel} = \frac{2}{\sqrt{3}}lU\left[\frac{3}{4}c_{\parallel} + \frac{1}{4}c_{\perp}\right], \quad \textbf{(E8)}$$

$$F_{\perp} = \frac{2}{\sqrt{3}}lU\left[\frac{1}{8}c_{\parallel} + \frac{7}{8}c_{\perp}\right], \quad \textbf{(E9)}$$

for motion parallel and perpendicular to the axis of the flagellum, respectively. This produces effective drag coefficients

$$C_{\parallel} = \frac{2}{\sqrt{3}}\left[\frac{3}{4}c_{\parallel}(l) + \frac{1}{4}c_{\perp}(l)\right], \quad \textbf{(E10)}$$

$$C_{\perp} = \frac{2}{\sqrt{3}}\left[\frac{1}{8}c_{\parallel}(l) + \frac{7}{8}c_{\perp}(l)\right], \quad \textbf{(E11)}$$

which, for clarity, give rise to drag forces acting on the flagella of axis length l travelling at speed U of

$$F_{\parallel} = lUC_{\parallel}, \quad \textbf{(E12)}$$

$$F_{\perp} = lUC_{\perp}. \quad \textbf{(E13)}$$

It should be noted that $C_{\perp}$ and $C_{\parallel}$ are technically functions of the axis length l. However, we find that over the fairly small range of values of l that we consider here, we can, to good approximation, set these coefficients to be constant by evaluating at, say, $l = 3.5\,\mu\mathrm{m}$. This gives

$$C_{\parallel} = 1.5970\,\mu, \quad \textbf{(E14)}$$

$$C_{\perp} = 2.1796\,\mu. \quad \textbf{(E15)}$$

This is a fairly important result, deviating significantly from the classical result $c_{\perp} \approx 2c_{\parallel}$.

We select the radius of the body to be such that it experiences the same drag as a prolate spheroid of semi-axes $0.5\,\mu\mathrm{m}$ and $1.25\,\mu\mathrm{m}$ (approximating the true geometry of *S. enterica*) travelling along its long axis. The resistance/mobility matrix for such a spheroid is easily calculated (5), giving an appropriate body radius $a = 0.65252\,\mu\mathrm{m}$. Note this slightly alters the diffusive properties of the swimmer, though only by a few percent.

Diffusion of a sphere is trivial to calculate, as a sphere in Stokes flow experiences a drag of $6\pi\mu a U$. Using our radius $a = 0.65252\,\mu\mathrm{m}$ and an approximate temperature of $T = 300\,K$, as well as noting a time step of $t = 0.0435\,s$ in between observation over which speed is calculated, we determine a diffusive speed of

$$U_{Diff} = \sqrt{\frac{6k_B T}{6\pi\mu a t}}, \quad \textbf{(E16)}$$

where k_B is the Boltzmann constant. This diffusive speed will ultimately produce excellent agreement with the data for the initial time step. However, it is significantly more than the speeds oberserved for later pre-critical speeds. To correct for this, we can include the effects of the flagella. Let us suppose for simplicity (which should nonetheless provide a decent approximation of reality) that there are 6 flagella of length l protruding from the spherical body with uniform distribution, akin to the six faces of a die. Therefore, the swimmer remains isotropic, but for any given swimming direction, its drag has increased by an amount

$$\Delta D = lU \left[2C_{\parallel} + 4C_{\perp}\right]. \quad \textbf{(E17)}$$

However, we wish to consider a more general number of flagella N (5 in our model) and so we will approximate the additional drag per unit speed as

$$D_f(l;N) = \frac{lN}{6}\left[2C_{\parallel} + 4C_{\perp}\right]. \quad \textbf{(E18)}$$

This increases the drag on the swimmer, thereby reducing its diffusive speed to

$$U_{Diff} = \sqrt{\frac{6k_BT}{\left[6\pi\mu a + D_f(l;N)\right]t}}. \quad \textbf{(E19)}$$

This decreases as l increases and gives better agreement with the data. Note that this estimate neglects hydrodynamic interactions between the flagella and the cell body, which is not expected to have a large effect. We continue to use the above expression post-criticality, even if swimming is seen to dominate diffusion throughout.

The bacteria swim in room temperature LB medium. The dynamic viscosity of LB medium is $1.83\,\mathrm{cP}$ when at a temperature of $37\ ^{\circ}\mathrm{C}$ (6). If we assume a similar relationship between viscosity and temperature for the LB medium as there is with water, then accounting for this gives a dynamic viscosity of approximately $2.61\,\mathrm{cP}$ at room temperature. In SI units, this gives us an estimate for the dynamic viscosity $\mu \approx 0.00261\,\mathrm{Pa\cdot s}$.

Applying Resistive Force Theory allows us to calculate consistent expressions for the driving force generated by the flagella in terms of either the angular rotation Ω and axis length l, or the motor torque τ.

$$F = \frac{c_{\perp} - c_{\parallel}}{2} Rl\Omega = \frac{c_{\perp} - c_{\parallel}}{3c_{\perp} + c_{\parallel}} \frac{\sqrt{3}\tau}{R}, \quad \textbf{(E20)}$$

where $R \approx 0.2\,\mu\mathrm{m}$ is the helix radius. Note we use the standard RFT coefficients, $c_{\perp}$ and $c_{\parallel}$, not the modified ones, $C_{\perp}$ and $C_{\parallel}$. The important consequence of this is that, assuming the molecular motors generate constant torques, the force generated by the flagella are constant (within particular assumptions required for RFT to be accurate, which are satisfied post-criticality) whilst the angular rotation rate decreases as the flagella grow. This also allows us to calculate τ in terms of F for comparison with pre-existing literature. The actual value of F is determined by numerically matching the theory to the data to minimise the square errors.

The value of the bending modulus EI varies greatly as different values of motor torque cause twisting of the hook, reconfiguring its molecular structure and causing stiffening as the torque increases (7, 8). We identify the critical length l_c in the same way as the force - by matching the theory to the data to minimise square errors. It is easy to do matching of l_c and F simultaneously as the value of l_c affects the value of l at which transition occurs, whilst F represents the size of the speeds achieved. This then allows us to calculate the torsional spring constant K and we then approximate the hook bending modulus by $EI \approx Kl_h$ where $l_h \approx 55\,\mathrm{nm}$ is the length of the hook (9).

Final Model. When $l < l_c$, the stable angle is $\theta = 0$ and the corresponding swimming speed is $U = 0$. For $l > l_c$, the stable angle is given by the non-zero solution to the equation

$$K = \frac{Fl_c^2 C_{\perp}}{\frac{12\pi\mu a}{N} + 2C_{\perp} l_c}, \quad \textbf{(E4)}$$

$$K\theta = \frac{Fl^2 C_{\perp}\sin(\theta)\cos(\theta)}{\frac{12\pi\mu a}{N} + 2l\left(C_{\parallel}\sin^2(\theta) + C_{\perp}\cos^2(\theta)\right)}. \quad \textbf{(E21)}$$

The swimming speed that results is then

$$U = \frac{2F\sin(\theta)}{\frac{12\pi\mu a}{N} + 2l\left(C_{\parallel}\sin^2(\theta) + C_{\perp}\cos^2(\theta)\right)}. \quad \textbf{(E22)}$$

We must incorporate into this the diffusive speed

$$U_{Diff} = \sqrt{\frac{6k_BT}{\left[6\pi\mu a + \frac{lN}{6}\left[2C_{\parallel} + 4C_{\perp}\right]\right]t}}. \quad \textbf{(E23)}$$

Finally, to compare with experimental data, which measured the swimming speed in a single plane, we must multiple by $\sqrt{2/3}$ to account for the reduced degrees of freedom. We obtain the final speed

$$U_{total} = \sqrt{\frac{2}{3}}\sqrt{U^2 + U_{diff}^2} \quad \textbf{(E24)}$$

Model: Predictions for the optimal range of k_{on} from the injection-diffusion model

In this section we review the derivation of the flagella growth law from the injection-diffusion model and detail the analysis leading to the predicted narrow range of k_{on} for optimal filament elongation shown in Figure 2 of the main text. Following Ref. (1), the density of flagellin monomers $u(x,t)$ at position x inside the flagella channel evolves according to a diffusion equation,

$$\frac{du}{dt} = D\frac{d^2u}{dx^2}, \tag{E25}$$

where D is the diffusion coefficient for the monomers. At the growing end, $x = L$, we assume monomers quickly polymerize onto the flagella, so that $u(L,t) = 0$. At the basal end, $x = 0$, the insertion rate for in empty filament is k_{on}, but insertion is decreased if the filament is not empty. Thus, the net flux of monomers per unit time inserted at the basal end is

$$J_0 = k_{\mathrm{on}}[1-u(0,t)] = -\frac{D}{l}\frac{du(x,t)}{dx}\bigg|_{x=0}, \tag{E26}$$

where l is the length of the extended flagellin monomer inside the channel. Note that the second equality comes from writing the diffusion equation, Eq. (E25), as the derivative of the flux, $du/dt = -l\,dJ_x/dx$ with $J_x = -(D/l)du/dx$. Since the elongation of the filament is slow compared to motion of individual monomers, we assume a quasisteady state for the monomer density, $d^2u/dx^2 = 0$ or J_x constant. Enforcing this condition leads to linear decay of the monomer density in the filament with constant slope $du/dx = -u(0)/L$. Combined with Eq. (E26), we obtain a relation between the filament length and basal end monomer density

$$u(0) = \frac{L}{D/(k_{\mathrm{on}}l)+L}. \tag{E27}$$

Finally, the growth rate of the filament is $dL/dt = \beta J_L$, where β is the increase in length due to polymerization of one monomer and $J_L = -(D/l)du/dx\big|_{x=L}$ is the monomer flux at the growing end of the filament. Since the flux is constant, we obtain

$$\frac{dL}{dt} = \frac{\beta D}{l}\frac{1}{D/(k_{\mathrm{on}}l)+L} = \frac{a}{b+L}, \tag{E28}$$

where we define $a = \beta D/l$ and $b = D/(k_{\mathrm{on}}l)$. Integrating this equation gives the growth curve for the filament,

$$L(t) = -b+\sqrt{b^2+2at}. \tag{E29}$$

We fix $\beta = 0.47\,\mathrm{nm}$, $l = 74\,\mathrm{nm}$ (1), and determine D and k_{on} by fitting to growth curves measured in the multilabeling experiments (Figure 3B). Fixing $D = 6\cdot10^{-13}\,\mathrm{m^2s^{-1}}$ to the fit value, we use the model to predict how the flagella growth depends on the injection rate k_{on}.

The red curve in Figure 2A shows the elongation L given in Eq. (E29) as a function of k_{on} for growth time $t = 20\,\mathrm{min}$. Solving for t we obtain the time to reach a particular flagella length,

$$t(L,k_{\mathrm{on}}) = \frac{L}{2a}(2b+L). \tag{E30}$$

The dependence of $t(L,k_{\mathrm{on}})$ on k_{on} is shown by the blue curve in Figure 2A for $L = 2.5\,\mu\mathrm{m}$, the minimal length required for motility.

To understand the improvements in performance associated with increasing k_{on}, we compute the gain in time to reach motility ($L = 2.5\,\mu\mathrm{m}$) from doubling k_{on},

$$\Delta t(k_{\mathrm{on}}) = t(k_{\mathrm{on}}) - t(2k_{\mathrm{on}}) = \frac{L}{2\beta k_{\mathrm{on}}}, \tag{E31}$$

which is shown in green in Figure 2B. Finally, we define the marginal cost to be the required increase in insertion rate Δk_{on} to reduce the time to reach motility by Δt seconds,

$$\Delta t(\Delta k_{\mathrm{on}}) = t(k_{\mathrm{on}}) - t(k_{\mathrm{on}}+\Delta k_{\mathrm{on}}). \tag{E32}$$

Solving for Δk_{on}, the marginal cost is

$$\Delta k_{\mathrm{on}} = \frac{k_{\mathrm{on}}^2\beta\Delta t}{L-k_{\mathrm{on}}\beta\Delta t}. \tag{E33}$$

The purple curves in Figure 2B show the marginal cost for a few different gains Δt.

Model: Arrival and insertion timescales and the associated energy cost

As described in the main text, the injection of flagellin into the flagella channel involves two timescales: arrival t_a (how long it takes a partially unfolded FliC to arrive at the flagella base) and insertion t_i (how long it takes for the pmf-powered complex to fully insert the FliC). These combine to set the overall on rate $k_{on} = (t_a + t_i)^{-1}$ for FliC. Energy is consumed in both the arrival and insertion process. Association with the chaperon FliS puts FliC in a partially unfolded state suitable for insertion; energy must be dissipated when the chaperon is removed before it arrives at the flagella base. The insertion complex then must overcome the free energy difference (including both entropic and enthalpic contributions) between the partially unfolded and nearly straight configurations. We will estimate each of the time scales t_a and t_i and their relation to the energy input for these functions.

Insertion time and energy cost. We model the insertion process using force balance between the drag force, driving force, and resistant entropic force. The former arises due to friction coefficient $\mu_0 l$ which is proportional to the current length of insertion l. The pmf driving force pushes the FliC into the channel with constant force f_d, while an entropic force f_r tends to push FliC out of the channel (where the protein has access to more configurations). Writing the driving force $f_d = G_0/\delta l$ in terms of the energy G_0 expended in one pmf cycle and the insertion distance δl per cycle, we have the following force balance equation,

$$\mu_0 l \frac{dl}{dt} = G_0/\delta l - f_r. \qquad \textbf{(E34)}$$

Solving for $l(t)$, we obtain

$$l(t)^2 = \frac{2t}{\mu_0}\left(G_0/\delta l - f_r\right). \qquad \textbf{(E35)}$$

The total insertion time is defined by $l(t_i) = L$ with $L = 74$ nm being the length of the entire extended flagellin protein. Solving for t_i, we find

$$t_i = \frac{\mu_0 L^2}{2\left(G_0/\delta l - f_r\right)}. \qquad \textbf{(E36)}$$

Finally, we express the total energy for insertion $E_i = (L/\delta l)G_0$ in terms of $k_{on} = (t_a + t_i)^{-1}$, treating t_a as a constant (since t_a is independent of the insertion energy cost),

$$E_i = \frac{\mu_0 L^3}{2t_i} + \Delta G = \frac{\mu_0 L^3 k_{on}}{2\left(1 - k_{on} t_a\right)} + \Delta G, \qquad \textbf{(E37)}$$

where $\Delta G = f_r L$ is the total entropy difference between the free floating and fully inserted FliC. Notice that t_a sets an upper bound $\max(k_{on}) = (t_a)^{-1}$ that is only exactly achieved with infinite dissipation by the pmf, $G_0 \to \infty$ (which leads to infinitely fast insertion, $t_i \to 0$).

To proceed, we estimate the drag constant from the diffusion coefficient for flagellin in the flagella channel that was fitted from the injection-diffusion model: $D = 0.6\,\mu\mathrm{m}^2/\mathrm{s}$, so that $\mu_0 = k_B T/(DL) = (2.3 \times 10^{-8}\,\mathrm{s/nm}^3) \times k_B T$. Because this drag coefficient is very small, the insertion process is very fast. The pmf energy per cycle is roughly $\sim 6 k_B T$ and we estimate the entropic energy cost for inserting by $\delta l \approx 1 - 2\,\mathrm{nm}$ to be around $\sim 2 - 4 k_B T$ (10), which is dominant compared to the enthalpic difference estimated in calorimetry experiments (11). Thus, the denominator in Eq. (E36) is $\mathcal{O}(k_B T)$ and the resulting insertion time $t_i \sim \mathcal{O}\left(10^{-4}\,\mathrm{s}\right)$. This calculation, using $L = 74\,\mathrm{nm}$, assumes the extended flagellin in the channel is alpha-helical (12, 13), but in a beta-strand conformation the chain would be about twice as long. The longer flagellin length leads to a slightly slower, but still rapid, insertion time $t_i \sim \mathcal{O}(10^{-3}\,\mathrm{s})$. In either scenario, compared to the measured $k_{on}^{-1} \approx 0.03\,\mathrm{s}$, we see that insertion is not the dominant timescale; spending more energy to decrease t_i has negligible impact on the overall insertion rate k_{on}. It is important that the pmf is strong enough to overcome the entropic force f_r, but once this is the case, the insertion step will be extremely fast.

It is also worth noting that the excess energy cost for this fast insertion is relatively small: $E_i/\Delta G = 1 + \mu_0 L^3/(2 t_i \Delta G) \approx 1.25$ for $\Delta G = 200 k_B T$ and $t_i = 10^{-4}\,\mathrm{s}$. The cell only spends $\sim 25\%$ more energy than if it executed the insertion adiabatically (infinitely slowly with minimal energy cost).

Arrival time and energy cost. From the analysis in the preceding section, we have shown that the insertion rate k_{on} is limited by the arrival of FliC at the insertion complex. We assume the arrival time t_a is diffusion limited and that once the FliC is within a distance d of the insertion complex (the "capture radius") it binds with probability 1 and is quickly inserted into the flagella channel as described above. The mean first passage time for a partially unfolded FliC to diffuse to the injection site is then,

$$t_a = \frac{1}{4\pi d D_c [C]_u}, \qquad \textbf{(E38)}$$

where D_c is the partial unfolded FliC diffusion constant in fluid (outside the flagella channel), and $[C]_u$ is the concentration of partially unfolded FliC. Note that $[C]_u$ is specifically the local concentration near the flagella base, where the binding to the insertion complex occurs. The concentration need not be uniform throughout the cell if the chaperon FliS is preferentially removed near the flagella base (either by the ATPase or some pmf-powered energy consuming mechanism). Increasing the dissipation rate can drive the system to a higher concentration of partially unfolded FliC, thereby reducing the arrival time t_a via Eq. (E38). If the concentration were uniform, the minimal arrival time is simply set by the total FliC concentration $[C]_0$, $t_{\min} = (4\pi d D_c [C]_0)^{-1}$. On the other hand, if the ATPase activity is localized near the flagella base, we have $t_{\min} = (4\pi d D_c [C]_0^{\mathrm{eff}})^{-1}$, where

$[C]_0^{\text{eff}} > [C]_0$ is some effective maximal concentration due to the localization of the ATPase/pmf activity. With this strategy, the cell can increase the local concentration above cell's average concentration $[C]_0$.

To model the energetics, we will use a simple two-state model (see main text, Fig. 4) for the populations of the chaperoned flagellin FliC-FliS and the free partially unfolded flagellin FliC, with concentrations $[C]_s$ and $[C]_u$ respectively with $[C]_0^{\text{eff}} = [C]_s + [C]_u$. There are two transitions between these states: (1) chaperon removal with rate k_{off} (with reverse rate k'_{off}) likely involving the ATPase or a pmf powered mechanism (e.g. interactions with the FlhA ring before the FliC reaches the export gate) and (2) FliC that is not injected can rebind to FliS, with rate k_s (and reverse rate k'_s). There are clearly sub-steps within this cycle, for example binding to the ATPase/FlhA or the FliC folding before binding to a new chaperon. However, because little is known about these mechanisms (and the associated reaction rates), we opt for the minimal coarse-grained model. Each of these reactions are highly irreversible so the FliC tend to follow the cycle clockwise between the two states.

The model has steady state concentrations,

$$\begin{aligned} p_s &= [C]_s / [C]_0^{\text{eff}} = \frac{k_s + k'_{\text{off}}}{k_{\text{tot}}} \\ p_u &= [C]_u / [C]_0^{\text{eff}} = \frac{k_{\text{off}} + k'_s}{k_{\text{tot}}}, \end{aligned} \tag{E39}$$

where $k_{\text{tot}} = k_s + k'_s + k_{\text{off}} + k'_{\text{off}}$. The dissipation rate of this system is given by,

$$\begin{aligned} \dot{S} &= \left(p_u k_s - p_s k'_s\right) \log\left(\frac{p_u k_s}{p_s k'_s}\right) \\ &\quad + \left(p_u k'_{\text{off}} - p_s k_{\text{off}}\right) \log\left(\frac{p_u k'_{\text{off}}}{p_s k_{\text{off}}}\right) \\ &= \frac{k_{\text{off}} k_s - k'_{\text{off}} k'_s}{k_{\text{tot}}} \log\left(\frac{k_{\text{off}} k_s}{k'_{\text{off}} k'_s}\right) \\ &= \frac{e^{E_s}\left(e^{\Delta G} - 1\right)}{e^{E_0} + e^{2E_s} + e^{E_0 + E_s} + e^{E_s + \Delta G}} k_s \Delta G, \end{aligned} \tag{E40}$$

where in the final line we introduce $E_s = \log k_s / k'_s = \log k'_{\text{off}} / k^0_{\text{off}}$ and $E_0 = \log k_s / k^0_{\text{off}}$. Here k^0_{off} is the "bare" rate for the chaperon removal pathway with no energy input. This rate is enhanced by the dissipative mechanism ΔG: $k_{\text{off}} = k^0_{\text{off}} \exp(\Delta G)$. All energy scales are expressed in units of $k_B T$. The dissipation per flagellin ejection is then equal to $E_{\text{a}} = \dot{S} t_a \approx \dot{S} / k_{\text{on}}$. We can similarly write the arrival time (and hence $k_{\text{on}} \approx t_a^{-1}$) in terms of the cycle dissipation ΔG by combining $t_a = t_{\text{min}} / p_u$ and Eq. (E39),

$$k_{\text{on}} = t_{\text{min}}^{-1} \left(1 + \frac{e^{E_s}\left(e^{E_s} + e^{E_0}\right)}{e^{E_0} + e^{E_s + \Delta G}}\right)^{-1}. \tag{E41}$$

Finally, eliminating ΔG, we express the energy cost per flagellin ejected directly in terms of the resulting injection rate k_{on},

$$\begin{aligned} E_a(k_{\text{on}}) &= k_s \left(t_{\text{min}} + e^{-E_s}\left(t_{\text{min}} - k_{\text{on}}^{-1}\right)\right) \\ &\quad \times \log\left(\frac{k_{\text{on}} t_{\text{min}}\left(e^{E_0} + e^{E_s}\right)}{1 - k_{\text{on}} t_{\text{min}}} + e^{E_0 - E_s}\right) \\ &\approx k_s t_{\text{min}} \log\left(\frac{e^{E_s} k_{\text{on}} t_{\text{min}}}{1 - k_{\text{on}} t_{\text{min}}}\right), \end{aligned} \tag{E42}$$

where in the final line we have taken the large E_s limit to make the approximation. Notice that the energy cost diverges logarithmically as $k_{\text{on}} \to t_{\text{min}}^{-1}$, it takes infinite energy to increase k_{on} toward this limiting value. Conversely, the energy cost vanishes at $k_{\text{on}} = \left[\left(1 + e^{E_s}\right) t_{\text{min}}\right]^{-1}$. This injection rate is achieved just by spontaneous dissociation of the FliS chaperone from FliC but is extremely small because this is a rare event, $E_s \gg 1$.

A plot of the energy is shown in the main text (Figure 4). Here we used the following parameters: $E_s = 20 k_B T$, $E_0 = 10 k_B T$, and $t_{\text{min}} = 0.01\,\text{s}$. The minimum time scale is roughly estimated using $D_c = 10\,\mu\text{m}^2/\text{s}$, $d = 1\,\text{nm}$, and $[C]_0 = 1000/\mu\text{m}^3$ leading to $t_{\text{min}} = 0.008\,\text{s}$. Fig. 4 in the main text also shows the efficiency $\eta = U/E_a$, where U is the measured swimming speed as a function of flagella length. To express swimming speed in terms of k_{on} we use the length after 30 mins of growth predicted by the injection-diffusion model fit to our measurements of flagella growth. The peak efficiency falls in the measured range $k_{\text{on}} = 20 - 50\,\text{s}^{-1}$ and the quantitative shape of this curve is quite robust in the $E_s \gg 1$ regime. For smaller E_s, the peak tends to move toward slightly smaller k_{on}.

Table S2. Key constant physical parameters used in the swimming model. These are calculated in this section, except for N, which is the average number of flagella observed in the experiments.

Parameter	Symbol	Value	Units
Fluid dynamic viscosity	μ	2.61×10^{-3}	Pa s
Body radius	a	6.5252×10^{-7}	m
Flagellar parallel drag coefficient	$C_{\parallel}$	4.168×10^{-3}	Pa s
Flagellar normal drag coefficient	$C_{\perp}$	5.689×10^{-3}	Pa s
Number of flagella	N	5	

Table S3. Key physical variables of the swimming model.

Parameter	Symbol	Units	Equation
Flagella length	l	m	
Flagella angle	θ		E21
Forwards swimming speed	U	m/s	E22
Diffusive speed	U_{diff}	m/s	E23
Total speed	U_{total}	m/s	E24

Table S4. Key physical results, obtained by least squares matching of the swimming model to experimental data, or calculated from such values.

Parameter	Symbol	Value	Units	Calculation method
Driving force (per flagellum)	F	1.094×10^{-12}	N	Matching with data
Critical length	l_c	2.508×10^{-6}	m	Matching with data
Hook spring constant	K	9.4612×10^{-19}	Nm	E4

Supplementary references

1. Thibaud T Renault, Anthony O Abraham, Tobias Bergmiller, Guillaume Paradis, Simon Rainville, Emmanuelle Charpentier, Călin C Guet, Yuhai Tu, Keiichi Namba, James P Keener, Tohru Minamino, and Marc Erhardt. Bacterial flagella grow through an injection-diffusion mechanism. *eLife*, 6, 2017. ISSN 2050-084X. doi:10.7554/eLife.23136.
2. Adrien Ducret, Ellen M. Quardokus, and Yves V. Brun. MicrobeJ, a tool for high throughput bacterial cell detection and quantitative analysis. *Nature Microbiology*, 1(7):1–7, June 2016. ISSN 2058-5276. doi:10.1038/nmicrobiol.2016.77.
3. Debasish Das and Eric Lauga. Computing the motor torque ofescherichia coli. *Soft Matter*, 14(29):5955–5967, 2018. doi:10.1039/c8sm00536b.
4. Nicholas C. Darnton, Linda Turner, Svetlana Rojevsky, and Howard C. Berg. On torque and tumbling in swimming escherichia coli. *Journal of Bacteriology*, 189(5):1756–1764, 2007. doi:10.1128/jb.01501-06.
5. Sangtae Kim and Seppo J. Karrila. *Microhydrodynamics: Principles and selected applications*. Dover, 2009.
6. Karolina Sklodowska, Pawel Debski, Jacek Michalski, Piotr Korczyk, Miroslaw Dolata, Miroslaw Zajac, and Slawomir Jakiela. Simultaneous measurement of viscosity and optical density of bacterial growth and death in a microdroplet. *Micromachines*, 9(5):251, 2018. doi:10.3390/mi9050251.
7. Kwangmin Son, Jeffrey S. Guasto, and Roman Stocker. Bacteria can exploit a flagellar buckling instability to change direction. *Nature Physics*, 9(8):494–498, 2013. doi:10.1038/nphys2676.
8. Ashley L. Nord, Anaïs Biquet-Bisquert, Manouk Abkarian, Théo Pigaglio, Farida Seduk, Axel Magalon, and Francesco Pedaci. Dynamic stiffening of the flagellar hook. *Nature Communications*, 13(1), 2022. doi:10.1038/s41467-022-30295-7.
9. T. Hirano, S. Yamaguchi, K. Oosawa, and S. Aizawa. Roles of FliK and FlhB in determination of flagellar hook length in Salmonella typhimurium. *J Bacteriol*, 176(17):5439–5449, Sep 1994. doi:10.1128/jb.176.17.5439-5449.1994.
10. Hong-Qing Xie and Cheng-Hung Chang. Chemical potential formalism for polymer entropic forces. *Communications Physics*, 2(1):24, 2019. doi:10.1038/s42005-019-0118-8.
11. Shinya Honda, Hatsuho Uedaira, Ferenc Vonderviszt, Shun ichi Kidokoro, and Keiichi Namba. Folding energetics of a multidomain protein, flagellin11edited by a. r. fersht. *Journal of Molecular Biology*, 293(3):719–732, 1999. ISSN 0022-2836. doi:https://doi.org/10.1006/jmbi.1999.3175.
12. Linda Turner, Alan S. Stern, and Howard C. Berg. Growth of flagellar filaments of Escherichia coli is independent of filament length. *Journal of Bacteriology*, 194(10):2437–2442, May 2012. ISSN 1098-5530. doi:10.1128/JB.06735-11.
13. Alan S. Stern and Howard C. Berg. Single-file diffusion of flagellin in flagellar filaments. *Biophysical Journal*, 105(1):182–184, July 2013. ISSN 1542-0086. doi:10.1016/j.bpj.2013.05.030.
